# Quantification of myocardial hemorrhage using T2* cardiovascular magnetic resonance at 1.5T with ex-vivo validation

**DOI:** 10.1186/s12968-021-00779-4

**Published:** 2021-09-30

**Authors:** Yinyin Chen, Daoyuan Ren, Xingmin Guan, Hsin-Jung Yang, Ting Liu, Richard Tang, Hao Ho, Hang Jin, Mengsu Zeng, Rohan Dharmakumar

**Affiliations:** 1 Biomedical Imaging Research Institute, Dept of Biomedical Sciences, Cedars-Sinai Medical Center, Suite 400, 8700 Beverly Blvd, Los Angeles, CA 90048 USA; 2 Department of Radiology, Zhongshan Hospital, Fudan University, Shanghai, 200032 China; 3 Department of Cardiology, Zhongshan Hospital, Fudan University, Shanghai, China; 4 Department of Bioengineering, University of California at Los Angeles, Los Angeles, CA USA; 5 Department of Radiology, The First Affiliated Hospital of China Medical University, Shenyang, 110001 China; 6 Academia Sinica, Taipei, Taiwan; 7 Department of Medical Imaging, Shanghai Medical School, Fudan University and Shanghai Institute of Medical Imaging, Shanghai, 200032 China

**Keywords:** Myocardial infarction, Hemorrhage, Iron, T2*, Mass spectrometry

## Abstract

**Background:**

T2* cardiovascular magnetic resonance (CMR) is commonly used in the diagnosis of intramyocardial hemorrhage (IMH). For quantifying IMH with T2* CMR, despite the lack of consensus studies, two different methods [subject-specific T2* (ssT2*) and absolute T2* thresholding (aT2* < 20 ms)] are interchangeably used. We examined whether these approaches yield equivalent information.

**Methods:**

ST elevation myocardial infarction (STEMI) patients (n = 70) were prospectively recruited for CMR at 4–7 days post revascularization and for 6-month follow up (n = 43). Canines studies were performed for validation purposes, where animals (n = 20) were subject to reperfused myocardial infarction (MI) and those surviving the MI (n = 16) underwent CMR at 7 days and 8 weeks and then euthanized. Both in patients and animals, T2* of IMH and volume of IMH were determined using ssT2* and aT2* < 20 ms. In animals, ex-vivo T2* CMR and mass spectrometry for iron concentration ([Fe]_Hemo_) were determined on excised myocardial sections. T2* values based on ssT2* and absolute T2* threshold approaches were independently regressed against [Fe]_Hemo_ and compared. A range of T2* cut-offs were tested to determine the optimized conditions relative to ssT2*.

**Results:**

While both approaches showed many similarities, there were also differences. Compared to ssT2*, aT2* < 20 ms showed lower T2* and volume of IMH in patients and animals independent of MI age (all p < 0.005). While T2* determined from both methods were highly correlated against [Fe]_Hemo_ (R^2^ = 0.9 for both), the slope of the regression curve for ssT2* was significantly larger as compared to aT2* < 20 ms (0.46 vs. 0.32, p < 0.01). Further, slightly larger absolute T2* cut-offs (patients: 23 ms; animals: 25 ms) showed similar IMH characteristics compared to ssT2*.

**Conclusion:**

Current quantification methods have excellent capacity to identify IMH, albeit the T2*of IMH and volume of IMH based on aT2* < 20 ms are smaller compared to ssT2*. Thus the method used to quantify IMH from T2* CMR may influence the diagnosis for IMH.

**Supplementary Information:**

The online version contains supplementary material available at 10.1186/s12968-021-00779-4.

## Introduction

In the setting of ST-segment elevation myocardial infarction (STEMI), several imaging markers for risk stratification have been proposed, including infarct size [1], myocardial salvage index [2], microvascular obstruction [3], as well as intramyocardial hemorrhage (IMH) [4]. IMH is one of the major complications associated with revascularized myocardial infarctions (MI) in the patients with STEMI [5]. Emerging evidence now supports the notion that IMH is associated with major adverse cardiovascular events [5–7]. IMH has also been shown to result in abnormal iron deposition within the MI zone [8, 9], which portends larger grey zone volume, late arrhythmogenic risk, prolonged inflammation, contributing to the negative prognosis in the post MI period [10–14]. Thus methods that can accurately identify hemorrhagic MIs from non-hemorrhagic MIs are expected to be important in the diagnosis of MI patients with hemorrhage and novel therapies to mitigate the negative effects of hemorrhage.

T2* CMR is the widely accepted method for noninvasive detection and quantification of IMH [15]. For this purpose, two different approaches are commonly used to quantify the volume of IMH and concentration of iron (1/T2*) within the MI territories, namely a subject-specific approach (based on mean-2SD criterion [16], hereinafter referred to as ssT2*) and an absolute-threshold approach (based on a T2* cut-off of below 20 ms [17], hereinafter referred to as aT2* < 20 ms). Although both approaches are used interchangeably, only ssT2* approach has been validated against invasive standards [8, 9, 16], with aT2* < 20 ms approach being directly adopted from the standards set in the analysis of global myocardial iron-overloading conditions such as thalassemia and hemochromatosis [18]. However, currently there is a lack of consensus between these two approaches in the field between these approaches as their relative performance in the setting of hemorrhagic MI has not been investigated [5].

Based on previous studies demonstrating that ssT2* derived estimates of mean T2* of IMH can be greater than 20 ms at 1.5T [8, 15], we hypothesized that the two approaches (ssT2* and aT2* < 20 ms) are likely to yield disparate estimates of mean T2* and IMH volume in the acute and chronic settings. To test our hypothesis, we performed cardiovascular magnetic resonance (CMR) studies in patients with hemorrhagic MI at 1.5T and quantified the T2* and IMH volume using ssT2* and the aT2* < 20 ms. To validate our findings in patients, we performed studies in a large animal MI model with and without IMH and evaluated the performance of the approaches relative mass spectrometry.

## Methods

### Patient studies

#### Study population

Studies were approved by Institutional Review Board and all patients gave written informed consent prior to enrollment. Seventy consecutive MI patients were prospectively enrolled between January 2018 and August of 2019. The primary inclusion criteria were patients with reperfused for STEMI with percutaneous coronary intervention (PCI); and the primary exclusion criteria were previous MI, arrhythmia, renal insufficiency, metallic prosthetic implant, and claustrophobia. All patients underwent CMR (details below) 4–7 days post PCI. Seven patients were excluded due to lack of CMR (n = 4) or the non-evaluable T2* maps (n = 3). Among the remaining 63 patients, 28 were identified to be non-hemorrhagic based on T2* CMR, and the remaining 35 patients had hemorrhagic MI. A fraction of the patients (n = 43; 18 non-hemorrhagic and 25 hemorrhagic) were followed up with CMR at 6–8 months (20 patients were lost to follow up or incomplete CMR scans). Refer to Additional file 1: Fig. S1 for additional details.

#### CMR in patients

CMR was performed in a 1.5T CMR system (Aera, Siemens Healthineers, Erlangen, Germany). Following shimming and scouting, slice-matched short-axis cine images, T2* maps, and late-gadolinium-enhancement (LGE) images, covering the full LV were acquired in that order. Typical Imaging parameters for cine balanced steady-state free precession (bSSFP) were TR/TE = 2.5/1.1 ms, flip angle = 50°, and generalized auto calibrating partial parallel acquisition with an acceleration factor of 2. T2*-maps were constructed from multi-gradient-recalled acquisitions: TR = 800 ms, 8 TEs = 2.2–14.8 ms with ∆TE = 1.8 ms, flip angle 18°, and bandwidth = 814 Hz/pixel. Segmented breath-held LGE images were acquired 10 min post-injection of 0.15 mmol/kg gadolinium contrast agent (Magnevist; Bayer Healthcare, Berlin, Germany) using segmented phase-sensitive inversion recovery (PSIR) reconstruction with gradient-recalled-echo readouts (TR/TE = 11/3.2 ms, TI = 300 ms, flip angle 25°, and bandwidth = 140 Hz/pixel). Voxel size for all acquisitions were 1.5 × 1.5 × 8 mm^3^.

### Animal studies

#### Animal preparation

According to the protocols approved by the Institutional Animal Care and Use Committee, canines (n = 20; female, 20–25 kg) were subject to reperfused MI through complete occlusion of the left anterior descending (LAD) coronary artery below the first diagonal for 3 h followed by reperfusion. Animals surviving the MI (n = 16) underwent CMR. A total of 10 canines imaged at 7 days after reperfusion for acute scan, and 8 weeks for chronic scan showed evidence for IMH, others were negative for IMH (n = 6). Following the 8-week CMR, animals were humanely euthanized, hearts were explanted and ex-vivo T2* CMR was performed (Refer to Additional file 1: Fig. S2 for additional details).

#### CMR in animals

CMR was performed in a 1.5T clinical CMR system (Espree, Siemens Healthineers). Slice-matched short-axis cines, T2* maps, and LGE images covering the full length of the left ventricle (LV) were acquired in that order. The scan parameters of cine bSSFP were: TR/TE = 3.5/1.3 ms, flip angle = 70°, and bandwidth = 930 Hz/pixel. Short-axis T2* maps were acquired with the following imaging parameters: TR = 240 ms, 6 TEs = 3.4–18.4 ms with ∆TE = 3.0 ms, flip angle 12°, voxel size = and bandwidth = 566 Hz/pixel. LGE images were acquired at least 10-minites post-injection of 0.2 mmol/kg gadolinium contrast agent (Magnevist; Bayer Healthcare) with PSIR reconstruction (TR/TE = 3.5/1.5 ms, TI = 300 ms, flip angle = 45°, and bandwidth = 1002 Hz/pixel). Voxel size for all acquisitions were 1.5 × 1.5 × 8 mm^3^. Ex-vivo 2D T2*-weighted images were acquired covering the LV with similar scan parameters as in vivo except for the slice thickness (ex vivo slice thickness = 5 mm).

#### Histology and inductively coupled plasma mass spectrometry

Mass spectrometry is the gold-standard for determining the iron concentration in tissue, and has been extensively used to validate T2* measures against iron concentration in the myocardium in ischemic and non-ischemic pathologies [8, 19, 20]. Hemorrhagic and remote myocardium were identified on the basis of ex-vivo T2* CMR (for identification of hemorrhage) and triphenyl tetrazolium chloride (TTC) staining (for identification of MI zones). Representative sections were acquired from tissue samples of infarcted and remote area from each animal, and stained with Perl’s staining to confirm hemorrhagic MIs. The remaining hemorrhagic sections were analyzed for iron concentration using a quadrupole-based X series 2 ICP-MS equipped with Collision Cell Technology (Thermo-Fisher Scientific, Waltham, Massachusetts, USA). The iron concentration ([Fe]_Hemo_) was calculated by weight-averaging the Fe content across all the hemorrhagic samples from each animal.

### Image analyses

All image analyses were performed using cvi^42^ (Circle Cardiovascular Imaging Inc., Calgary, Alberta, Canada) by a radiologist with 6 years of experience in CMR. MI territories were identified on LGE images using the mean-5SD approach [21], and the regions of microvascular obstruction (MVO) were manually included within the zone of MI. In patients, LV end-diastolic volume (LVEDV), LV end-systolic volume (LVESV) and LV ejection fraction (LVEF) were computed based on cine CMR and were used to compute the percentage change in ΔLVEDV and ΔLVEF between acute and chronic phases.

#### Quantification of mean T2* and volume of IMH

IMH zones were identified as hypointense cores within MI on the T2*-weighted maps at the longest TE (patients: TE = 14.8 ms, and canines: TE = 18.4 ms). Mean T2* and volume of IMH were determined using ssT2* and aT2* < 20 ms approaches. For ssT2*, a reference region of interest (ROI) was drawn in the remote myocardium on T2*-weighted images, and the regions with mean signal intensity below 2SD of the reference ROI were defined as Hemo_ssT2*_. For aT2* < 20 ms, regions with T2* < 20 ms on T2* maps were defined as Hemo_aT2*<20 ms_. For the in-vivo T2* analysis, care was taken not to include myocardial regions affected by off-resonance artifacts. Per-slice and whole-heart T2* values of IMH territories were determined from the in-vivo images acquired in the acute and chronic phases both for patients and animals, as well as ex-vivo images. Similarly, IMH extent on per-slice basis and IMH volume on whole-heart basis were determined from image analyses performed on in-vivo and ex-vivo images in animals with and without hemorrhage. Absolute differences in T2* value of IMH, and IMH volume were determined between Hemo_ssT2*_ and Hemo_aT2*<20 ms_ and were labeled as ΔT2*, and Δvolume (%LV), respectively. The relative differences were then calculated as the ratio of absolute differences to the value of ssT2*, and labeled as Relative ΔT2*(%) and Relative Δvolume (%).

#### T2* cut-offs for ssT2* and aT2* approaches vs. [Fe]_Hemo_

Standard ssT2* (with mean-2SD criterion) and aT2* < 20 ms were applied to ex-vivo T2* CMR data and the resulting T2* values were regressed against [Fe]_Hemo_ from mass spectroscopic measurements. In addition, to assess whether the different cut-offs for each of the methods could potentially improve the quality of the regressions, additional cut-offs for both methods were tested (mean-3SD and mean-4SD for ssT2* approach; and 15 ms, and 25 ms for absolute thresholding approach) and the resulting T2* values of IMH territories were regressed against [Fe]_Hemo_.

#### Determination of optimized absolute thresholds for aT2* approach relative to ssT2*

Various T2* cut-offs were evaluated for identifying the presence of IMH and quantification of IMH volume. In addition to the cut-off of 20 ms, cut-offs of 5 ms to 30 ms with 5-ms increments were used successively for measuring the IMH volumes in patients and animals. Once the closest upper and lower bounds of T2* cut-offs were determined within the 5-ms increments, additional analyses with more subtle thresholds incremented by 1-ms were evaluated. At the segmental level, a 16-segment American Heart Association model, excluding the apex was used. Based on the evidence of more favorable relation between ex-vivo T2* and [Fe]_Hemo_ with ssT2*, ssT2* was used as the reference standard for receiver-operating characteristic analysis. Each segment was dichotomized as either positive or negative for IMH based on the criterion of at least 5% hypointense area within MI on ssT2* approach. Subsequently, sensitivity and specificity at the different thresholds (range 5–50 ms) were determined and used to construct the Receiver Operating Characteristics (ROC) curves and to identify the optimal aT2* cutoffs.

### Statistical analyses

All statistical analyses were conducted using SPSS (version 20.0, Statistical Package for the Social Sciences, International Business Machines, Inc., Armonk, New York, USA). Continuous variables determined to be normal are reported as mean ± standard deviation (SD); otherwise they are reported as median and interquartile range (IQR). Categorical variables are reported as numbers, along with relative values as percentages. The normality test of continuous variables was assessed by Kolmogorov–Smirnov test. The absolute and relative differences between the two approaches were evaluated using paired Student’s *t-*test or one sample *t*-test. Association between continuous parameters was assessed using Pearson correlation coefficients and were compared using *cocor* package [22]. The comparison of slopes of regression lines was performed using general linear model to determine whether the approaches and variables have an interaction. Differences between two approaches were also illustrated by using a Bland–Altman plot. The inter- and intra-observer reliability in measuring IMH T2* were assessed by two independent readers using intraclass correlation coefficient (ICC). Comparison of IMH volumes from various T2* cutoffs was conducted using repeated measures of analysis of variance. Least-significant difference (LSD) test was used to perform multiple comparisons. ΔLVEDVI and ΔLVEF were regressed against acute IMH volume and T2*. ROC analysis was used to compute the area-under-the-curve (AUC) using ssT2* as the reference standard, and AUC comparisons were made with approach proposed by DeLong et al. [23]. All tests were two-tailed, and a p-value of < 0.05 was used to determine statistical significance.

## Results

Patient characteristics along with CMR findings (LVEF, MI volume, MVO volume) are summarized in Table 1. In patient studies, slices demonstrating evidences of both LGE and hemorrhage were chosen, and 5 acute and 3 chronic slices were excluded due to artifacts. Thus, 236 acute and 128 chronic 2D T2* maps were available for final analysis. For segmental analysis, after excluding MI segments affected by off-resonance artifacts (69 acute and 86 chronic MI segments), 361 acute MI segments (hemorrhagic 184, non-hemorrhagic 177) and 178 chronic MI segments (hemorrhagic 84, non-hemorrhagic 94) were available for segmental analysis. From canine studies, slices having both LGE and hemorrhage were chosen, and 2 acute and 2 chronic slices were excluded due to artifacts. Thus 40 acute, 44 chronic and 55 ex-vivo 2D T2* maps were available for final analysis. For segmental analysis, after excluding 43 acute and 35 chronic MI segments due to off-resonance artifacts, 59 acute MI segments (29 hemorrhagic, 30 non-hemorrhagic) and 67 chronic MI segments (29 hemorrhagic, 38 non-hemorrhagic) were available for analysis. All animals identified to be hemorrhagic in the acute phase of MI showed evidence of iron within MI and absence of iron in the remote myocardium on Perl’s staining of ex-vivo sections.

**Table 1:** Characteristics of Patients Study Cohort

	Acute Phase (n = 63)	Chronic Phase (n = 43)
Age	56 ± 7	55 ± 8
Male sex, n (%)	56 (89)	38 (88)
Body mass index (kg/m^2^)	25.5 ± 2.5	25.7 ± 2.6
Cardiovascular risk factors, n (%)		
Hypertension	31 (49)	22 (51)
Diabetes	12 (19)	6 (14)
Hyperlipidemia	27 (43)	19 (44)
Smoking	40 (63)	29 (67)
Heart rate (bpm)	74 ± 14	70 ± 12
Infarct-related artery, n (%)		
Left anterior descending	38 (60)	27 (63)
Left circumflex	7 (11)	5 (12)
Right coronary artery	18 (29)	11 (25)
Cardiovascular magnetic resonance findings		
LV ejection fraction (%)	43.8 ± 7.2	46.0 ± 6.9
LV end-diastolic volume index (ml/m^2^)	87.8 ± 17.7	89.0 ± 16.2
LV end-systolic volume index (ml/m^2^)	50.5 ± 14.6	48.7 ± 13.2
Infarct volume (%LV)	33.3 ± 12.4	22.7 ± 8.6
Late MVO volume (%LV)	6.5 ± 6.0	–
Medication during admission, n (%)		
Antiplatelet therapy	63 (100%)	
Beta blocker	59 (94%)	
ACEI	53 (84%)	
ARB	16 (25%)	
CCB	5 (8%)	
Diuretic	24 (38%)	
Statin	63 (100%)	
Amiodarone	7 (11%)	
Nitrate	56 (89%)	

Data are reported as mean ± SD, median (IQR), or n (%) as appropriate;

None of the patients received thrombolysis

*ACEI* angiotensin converting enzyme inhibitor, *ARB* angiotensin receptor blocker, *CCB* calcium channel blocker, *LV* left ventricle, *MVO* microvascular obstruction

### Case examples

**Fig. 1:**
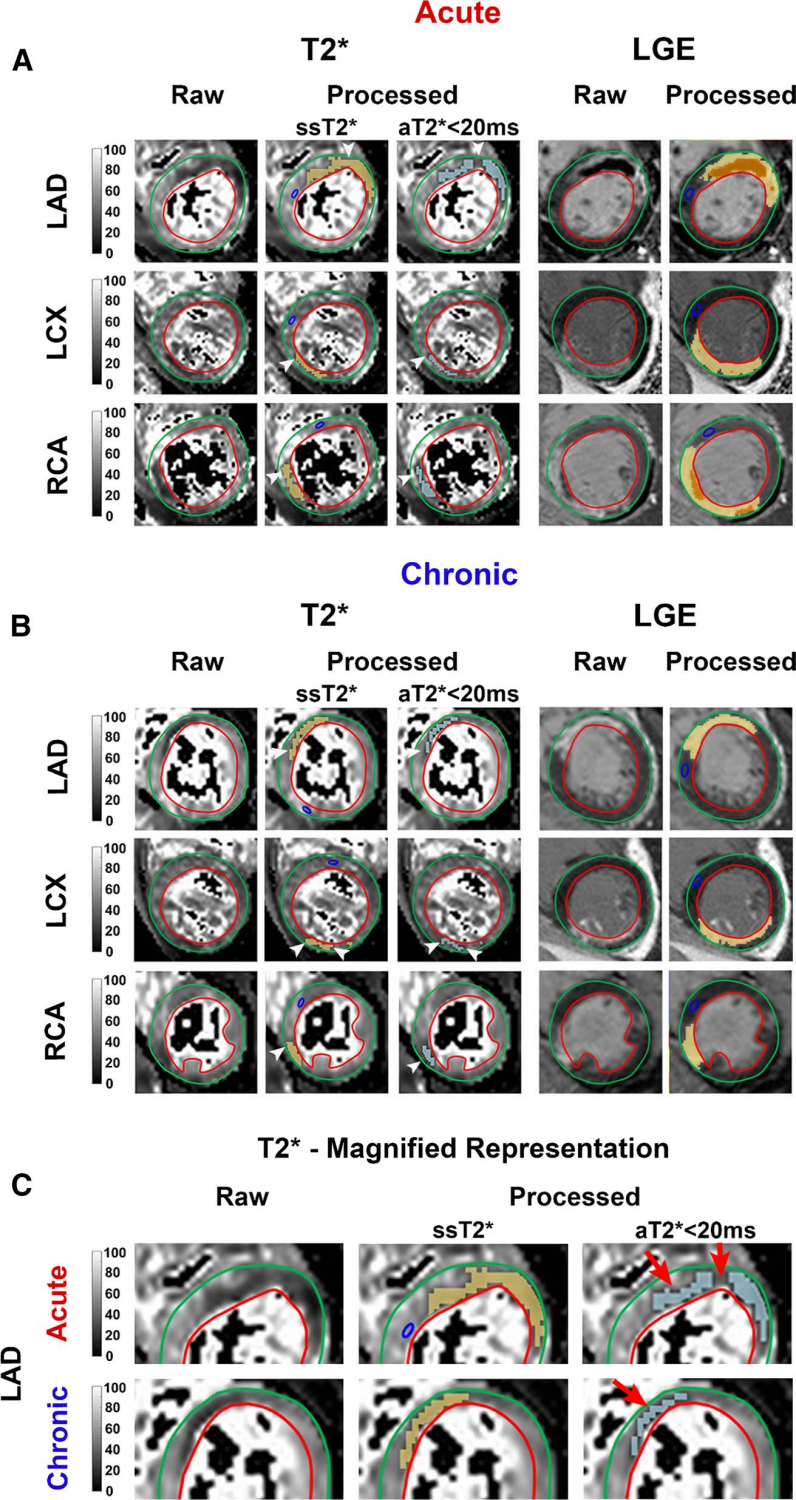
Representative Findings in Patients—Subject-Specific T2* (ssT2*) vs. 20-ms Absolute T2* (aT2* < 20 ms) Cut-off for Hemorrhage Characterization. Representative 2D short-axis T2*-weighted and T2* image sets, along with late-gadolinium-enhanced (LGE) CMR, acquired at 1.5T in patients with hemorrhagic myocardial infarction (MI) in the left anterior descending coronary artery (LAD), left circumflex coronary artery (LCX) and right coronary artery (RCA) territories in the acute (**A**) and chronic (**B**) myocardial infarction (MI) phases are shown. Both raw and processed image sets [using ssT2* (second column of panels **A** and **B**) and aT2* < 20 ms (third column of panels **A** and **B**)] are shown side-by-side for comparison. Magnified representation of T2* maps for the LAD infarction in panels **A**, **B** are shown in panel **C**. Note that although the location of intramyocardial hemorrhage (IMH) is the same with both ssT2* and aT2* < 20 ms, the extent of IMH identified using aT2* < 20 ms approach are visually smaller (see red arrows) compared to that determined using ssT2*

*Patients* Fig. 1 (Central illustration) shows representative 2D T2* images in patients with hemorrhagic MI in the LAD, left circumflex coronary artery (LCX) and right coronary artery (RCA) territories in the acute and chronic (follow-up) phases. IMH territories identified using ssT2* and aT2* < 20 ms show that compared to ssT2*, the regions identified using aT2* < 20 ms were not different in location but are visually smaller in extent.

**Fig. 2:**
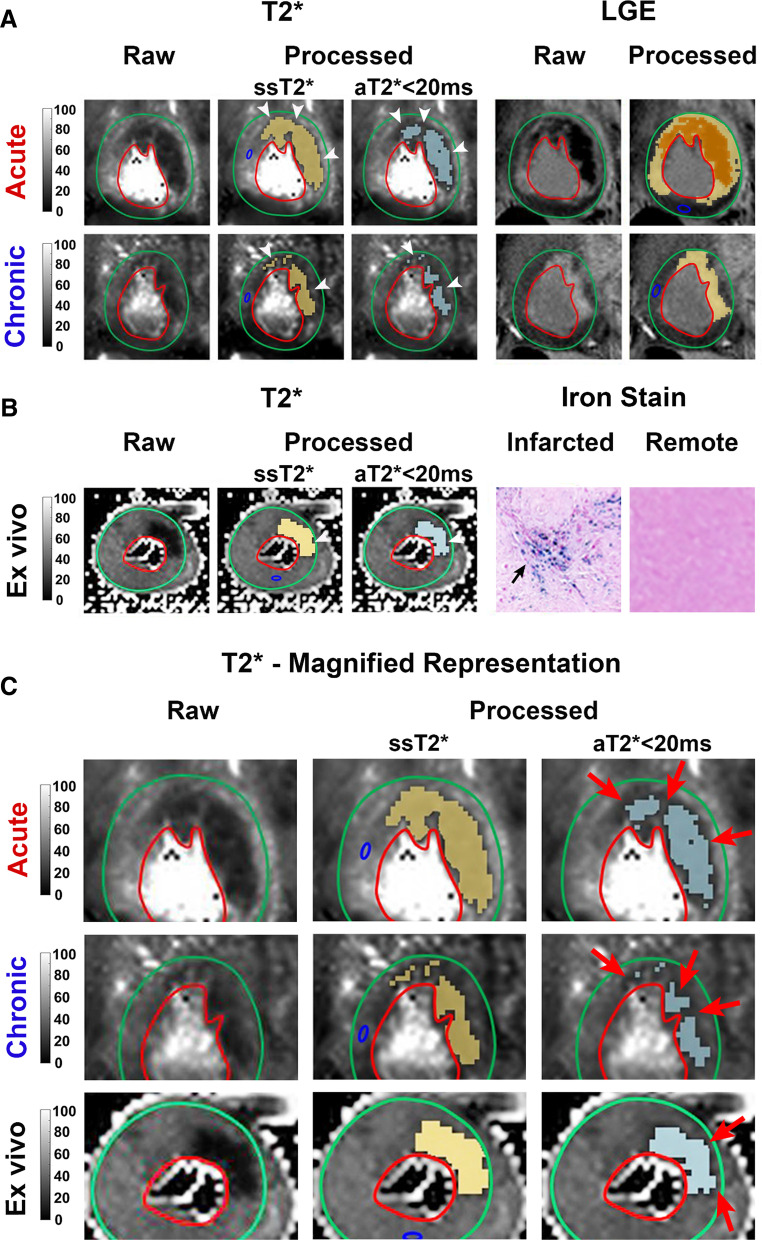
Representative Findings in Canines—Subject-Specific T2* vs. 20-ms Absolute T2* Cut-off for Hemorrhage Characterization. Representative T2* maps and corresponding LGE acquired at 1.5T in a canine with hemorrhagic MI in the LAD territory in the acute and chronic phases **A**. Ex-vivo T2* maps, along with histological validation of IMH are shown in panel **B**. Perl’s stain showed the presence of the iron deposition in the infarcted myocardium (arrow) which is not evident in the remote territory. In panels **A**, **B** both raw and processed image sets [using ssT2* (second column) and aT2* < 20 ms (third column)] are shown side-by-side for comparison. Panel **C** shows magnified representation of T2 maps from panels **A**, **B**. Note that similar to patients, although the location of IMH is the same with both ssT2* and aT2* < 20 ms, the extent of IMH identified using aT2* < 20 ms approach are visually smaller (see red arrows) compared to that determined using ssT2*

*Animals* Fig. 2 shows representative 2D T2* images acquired from a canine with reperfused hemorrhagic MI in the LAD territory in the acute and chronic phases, as well as, post-sacrifice (ex vivo). Similar to the findings in patients, IMH territories identified with aT2* < 20 ms were smaller in extent compared to ssT2* using mean-2SD.

### IMH quantification: subject-specific vs. absolute T2* thresholds

**Fig. 3:**
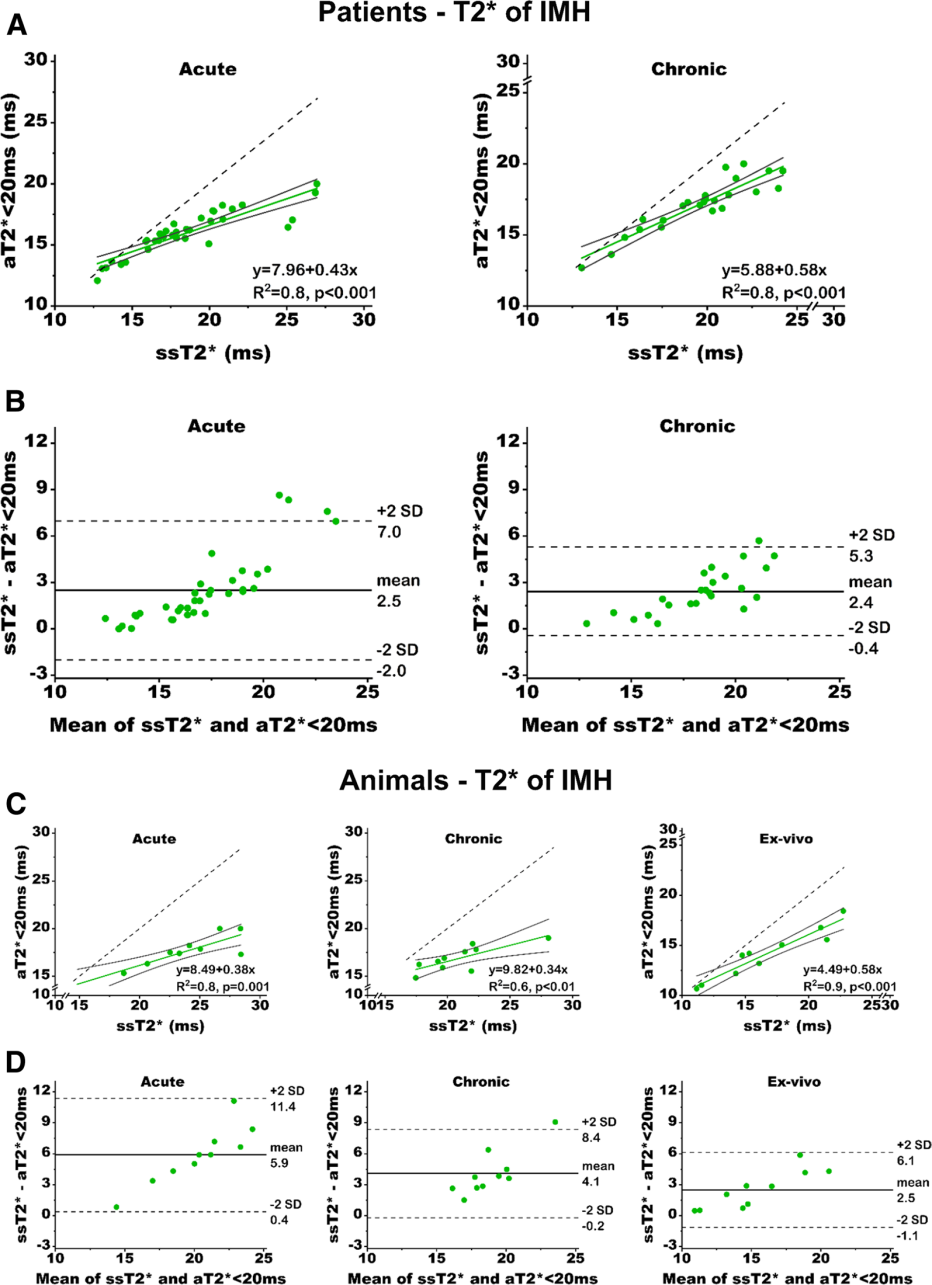
Linear Regression Analysis and Bland–Altman Plot for T2* of IMH Determined Using ssT2* and aT2* < 20 ms. **A** Linear regression analysis in patients (left—acute; right—chronic) were strongly correlated but the absolute T2* values deviated significantly (slopes < 1.0, both p < 0.001), which was also shown by Bland–Altman plot (**B**). **C** Linear regression analysis in animals at acute (left—acute; middle—chronic; and right—ex-vivo) show similar strong regressions and highly discordance T2* values as evidenced by the lines of best fit having slopes < 1.0 (all p ≤ 0.001). **D** shows Bland–Altman plot. The black solid lines denote the 95% confidence bands, and for reference the dotted line denoting the line of identify is shown

**Fig. 4:**
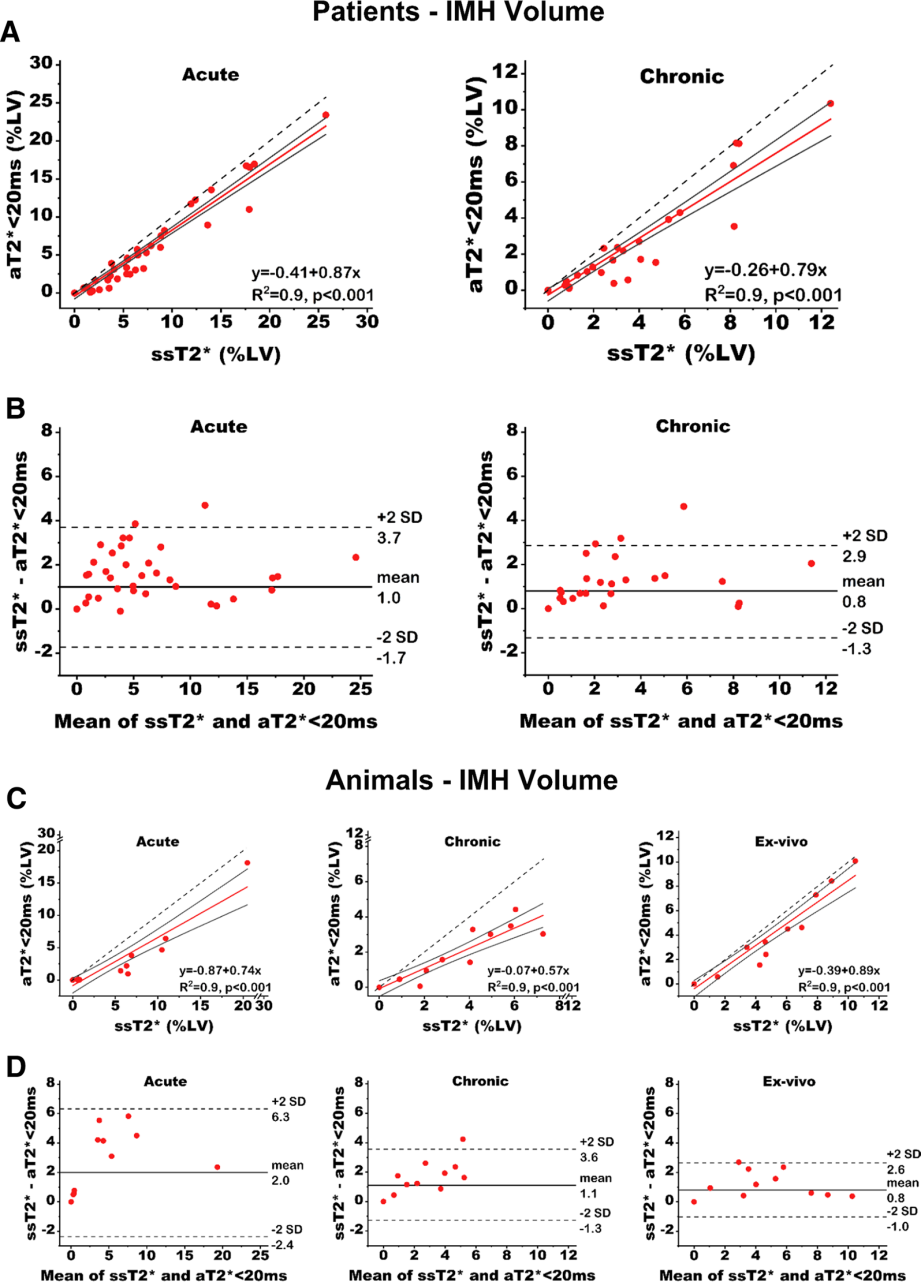
Linear Regression Analysis and Bland–Altman Plot for IMH Volume Determined Using ssT2* and aT2* < 20 ms. **A** Linear regression analysis in patients (left—acute; right—chronic MI) were strongly correlated but the IMH volumes deviated significantly (slopes < 1.0, both p < 0.001), which was also shown by Bland–Altman plot (**B**). **C** Linear regression analysis in animals at acute MI (left—acute; middle—chronic; and right—ex-vivo) show similar strong regressions and highly discordance IMH volumes as evidenced by the lines of best fit having slopes < 1.0 (all p < 0.05). **D** shows Bland–Altman plot. The black solid lines denote the 95% confidence bands, and for reference the dotted line denoting the line of identify is shown

*Patients* T2* values of IMH territories were well correlated (R^2^ = 0.8, p < 0.001 (acute), and R^2^ = 0.8, p < 0.001 (chronic), Fig. 3A). The slopes of the regression between aT2* < 20 ms and ssT2* were 0.43 [95% confidence interval: 0.33–0.52, p < 0.001 (vs. 1.0)] in the acute phase, and 0.58 [95% confidence interval: 0.47–0.67, p < 0.001 (vs. 1.0)] in the chronic phase, indicating that the dynamic range of T2* with ssT2* is markedly greater than aT2* < 20 ms. Notably, compared to ssT2* approach, aT2* < 20 ms approach underestimated the T2* of IMH territory (acute: mean bias of 2.5 ms, p < 0.001; chronic: mean bias of 2.4 ms, p < 0.001, Table 2). Similarly, the IMH volume quantified using the two approaches were also highly correlated (R^2^ = 0.9, p < 0.001, for both acute and chronic phases, Fig. 4A) but IMH volumes based on aT2* < 20 ms were significantly underestimated [(acute: mean bias of 1.8%LV, p < 0.001; and mean relative ∆volume of 32.7%, p < 0.001) and (chronic: mean bias of 1.3% LV, p < 0.001; and mean relative ∆volume of 42.8%, p < 0.001)] compared to ssT2 (see Table 2). The slopes of the regression curves between aT2* < 20 ms and ssT2* with respect to IMH volume in the acute phase was 0.87 [95% confidence interval: 0.77–0.92, p < 0.001 (vs. 1.0)], and in the chronic phase was 0.79 [95% confidence interval: 0.59–0.89, p < 0.001 (vs. 1.0)] respectively (see Fig. 4A). The intercepts of the regression were − 0.41 (p < 0.05) in the acute phase, and − 0.26 (p = 0.12) in the chronic phase.

**Table 2:** ssT2* vs. aT2* < 20 ms for whole-heart intramyocardial hemorrhage (IMH) T2* and Volume (%LV) in Patients and Canines with IMH

	Patients		Canines		
	Acute	Chronic	Acute	Chronic	Ex vivo
IMH T2* value (ms)					
ssT2*	18.4 ± 3.7	19.6 ± 3.0	23.3 ± 4.6	20.9 ± 3.0	16.6 ± 4.1
aT2* < 20 ms	15.9 ± 1.8	17.1 ± 1.9	17.4 ± 2.0	16.9 ± 1.3	14.1 ± 2.5
∆T2* (ms)	2.5 ± 2.3^‡^	2.4 ± 1.5^‡^	5.9 ± 3.0^‡^	4.1 ± 2.2^‡^	2.5 ± 1.9^†^
Relative ∆T2*(%)	11.9 ± 8.6^‡^	11.7 ± 6.0^‡^	23.7 ± 9.1^‡^	18.7 ± 7.2^‡^	13.5 ± 8.0^‡^
Remote T2* value (ms)	35.5 ± 3.3	33.0 ± 2.9	40.0 ± 6.5	34.6 ± 5.9	34.4 ± 5.6
IMH volume (%LV)					
ssT2*	8.0 ± 5.9	4.0 ± 3.1	6.9 ± 6.4	4.0 ± 2.1	5.9 ± 2.7
aT2* < 20 ms	6.2 ± 5.8	2.7 ± 2.9	3.8 ± 5.8	2.2 ± 1.5	4.6 ± 3.1
∆volume (%LV)	1.8 ± 1.4^‡^	1.3 ± 1.1^‡^	3.2 ± 2.1^†^	1.8 ± 1.1^‡^	1.3 ± 0.9^†^
Relative ∆volume (%)	32.7 ± 26.2^‡^	42.8 ± 27.2^‡^	67.5 ± 27.6^‡^	49.5 ± 21.4^‡^	28.7 ± 22.7^†^

aT2* absolute T2* < 20 ms; *IMH* intramyocardial haemorrhage, ssT2* subject specific T2*

^†^Indicates p < 0.005, ^‡^p < 0.001

*Animals* T2* of IMH territories were well correlated (R^2^ = 0.8, p = 0.001 (acute), R^2^ = 0.6, p < 0.01(chronic), and R^2^ = 0.9, p < 0.001 (ex-vivo), Fig. 3B). The slopes of the regression between aT2* < 20 ms and ssT2* were 0.38 [95% confidence interval: 0.14–0.54, p < 0.001 (vs. 1.0)] in the acute phase, 0.34 [95% confidence interval: 0.05–0.70, p = 0.001 (vs. 1.0)] in the chronic phase, and 0.58 [95% confidence interval: 0.40–0.69, p < 0.001 (vs. 1.0)] ex-vivo, indicating that the dynamic range of T2* with ssT2* is markedly greater than aT2* < 20 ms. Notably, similar to our findings in patients, compared to ssT2* approach, aT2* < 20 ms approach underestimated the T2* of IMH territory (acute: mean bias of 5.9 ms, p < 0.001; chronic: mean bias of 4.1 ms, p < 0.001; ex-vivo: mean bias of 2.5 ms, p < 0.005, Table 2). Similarly, the IMH volume quantified using the two approaches were also highly correlated (R^2^ = 0.9, p < 0.001, both in-vivo and ex-vivo, Fig. 4B) but IMH volumes based on aT2* < 20 ms were significantly underestimated [(acute: mean bias of 3.2%LV, p < 0.005; and mean relative ∆volume of 67.5%, p < 0.001), (chronic: mean bias of 1.8% LV, p < 0.001; and mean relative ∆volume of 49.5%, p < 0.001) and (ex-vivo: mean bias of 1.3% LV, p < 0.005; and mean relative ∆volume of 28.7%, p < 0.005)] compared to ssT2 (see Table 2). The slopes of the regression curves between aT2* < 20 ms and ssT2* with respect to IMH volume in the acute phase was 0.74 [95% confidence interval: 0.37–0.88, p < 0.005 (vs. 1.0)], in the chronic phase was 0.57 [95% confidence interval: 0.44–0.72, p < 0.001 (vs. 1.0)], and ex-vivo was 0.89 [95% confidence interval: 0.73–0.96, p = 0.05 (vs. 1.0)] respectively (see Fig. 4B).

### Inter-observer and intra-observer variability: subject-specific vs. absolute T2* thresholds

There was good to excellent agreement in quantifying IMH with ssT2* and aT2* < 20 ms approaches both in patients and animals in the acute and chronic phases of MI. Both inter- and intra-observer variabilities across species and infarct age showed intra-class correlation of > 0.85 (See Table 3).

**Table 3:** Inter- and intra-observer variability in IMH T2* measurement with ssT2* and aT2* < 20 ms

	Inter-observer		Intra-observer	
	ssT2*	aT2* < 20 ms	ssT2*	aT2* < 20 ms
Patients				
Acute	0.907 (0.602–0.983)	0.938 (0.373–0.990)	0.926 (0.635–0.987)	0.945 (0.714–0.990)
Chronic	0.859 (0.430–0.974)	0.884 (0.523–0.978)	0.875 (0.454–0.977)	0.912 (0.561–0.984)
Canines				
Acute	0.928 (0.530–0.992)	0.943 (0.645–0.993)	0.944 (0.614–0.993)	0.955 (0.645–0.995)
Chronic	0.882 (0.248–0.987)	0.903 (0.583–0.982)	0.910 (0.408–0.990)	0.936 (0.701–0.988)
Ex vivo	0.957 (0.644–0.995)	0.966 (0.776–0.996)	0.964 (0.763–0.996)	0.981 (0.856–0.998)

Values are reported as intraclass correlation coefficient with 95% confidence interval

### Ex-vivo validation with mass spectrometry

**Fig. 5:**
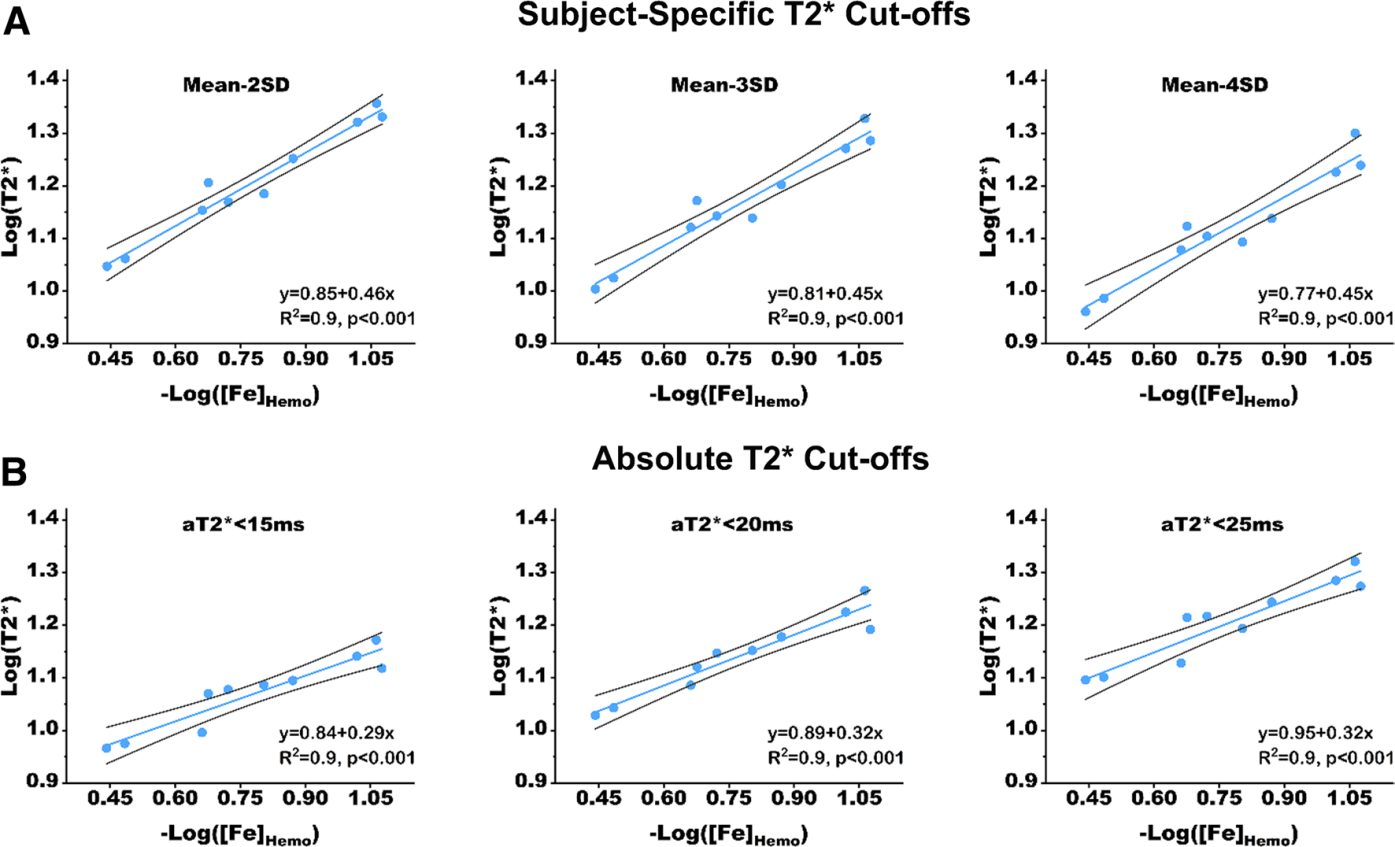
Relation between T2* and Iron Concentration Determined Using ssT2* and Absolute T2* with Various Cut-offs. **A** T2* and iron concentration ([Fe]_Hemo_) determined with ssT2* at cut-offs of mean-2SD, mean-3SD and mean-4SD. **B** T2* and iron concentration determined with absolute T2* at cut-offs of 15 ms, 20 ms and 25 ms. Note that both ssT2* and absolute T2* approaches yield a high correlation between log (T2*) and -log ([Fe]_Hemo_). However, note that the slope of the absolute T2* approach is markedly lower compared to ssT2* approach, regardless of the threshold, which supports the notion that the absolute T2* has a lower sensitivity compared to ssT2* approach for detecting changes in iron concentration. The regression lines are shown in blue and 95% confidence are shown in black

The Pearson correlation coefficients were not different between subject-specific and absolute-threshold based approaches (R^2^ = 0.9 for all cases, Fig. 5). When correlation coefficients were compared within the two approaches, no significant differences were observed (all p > 0.05). However, the slopes of the regression curves within ssT2* approach (mean-2SD: 0.46 (95% CI: 0.39–0.51); mean-3SD: 0.45 (95% CI: 0.38–0.51); mean-4SD: 0.45 (95% CI: 0.36–0.53), see Fig. 5A) were all significantly larger as compared to that within absolute-threshold based approach (aT2* < 15 ms: 0.29 (95% CI: 0.17–0.37); aT2* < 20 ms: 0.32 (95% CI: 0.21–0.40); aT2* < 25 ms: 0.32 (95% CI: 0.24–0.39), Fig. 5B). This supports the notion that the absolute thresholds have lower sensitivity for identifying IMH compared to ssT2* approaches. Further, the largest slope for the ssT2* was found with mean-2SD and aT2* < 20 ms for the absolute-thresholding approach, which provides additional validation for the current cut-offs used for the respective approaches. Refer to Additional file 1: Table S1 for additional details.

### Absolute T2* thresholds for optimal characterization of IMH

**Fig. 6:**
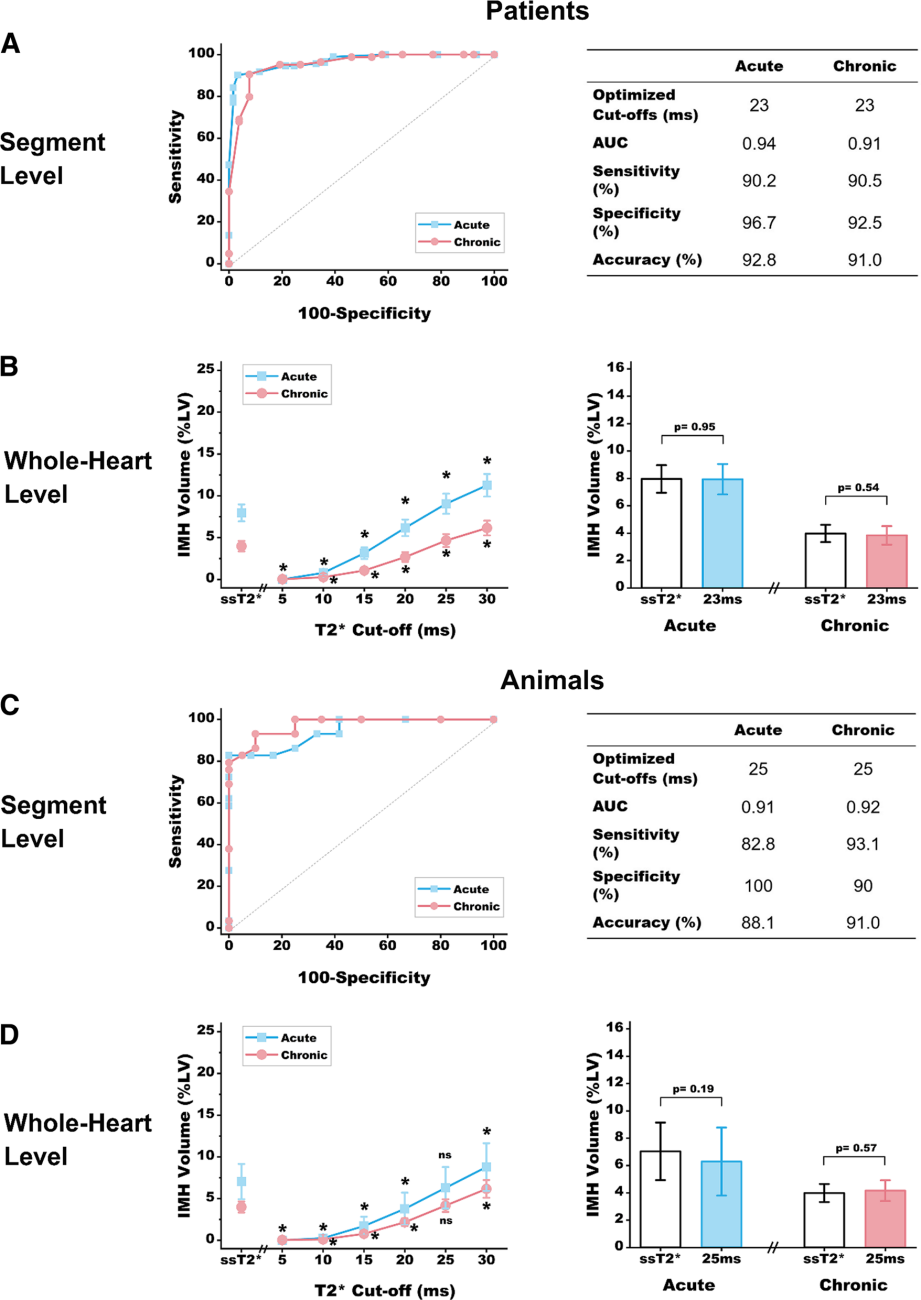
Optimization of Absolute T2* Cut-offs for Detecting IMH and Quantifying IMH volume. ROC curves for IMH detection based on segmental analysis of T2* images obtained in the acute and chronic phases using absolute T2* cut-offs in patients are shown in **A** left panel. **A** right panel shows optimized T2* cut-off with corresponding area under the cure (AUC), sensitivity, specificity and accuracy. **B** left panel shows the IMH volume detected at the whole-heart level at the various absolute T2* thresholds of 5–30 ms (in increments of 5 ms) and ssT2*. **B** right panel shows IMH volume detected at the whole-heart level in the acute and chronic phases based on optimized absolute T2* cut-off identified in **A** relative to ssT2*. ROC curves for IMH detection based on segmental analysis of T2* images obtained in the acute and chronic phases using absolute T2* cut-offs in animals are shown in **C** left panel. **C** right panel shows optimized T2* cut-off with corresponding area under the cure (AUC), sensitivity, specificity and accuracy. **D** left panel shows the IMH volume detected at the whole-heart level at the various absolute T2* thresholds of 5–30 ms (in increments of 5 ms) and ssT2*. **D** right panel shows IMH volume detected at the whole-heart level in the acute and chronic phases based on optimized absolute T2* cut-off identified relative to ssT2* from segmental analysis in panel (**C**). *Indicates statistical significance (p < 0.05) compared to ssT2* approach, and ns represents no statistical significance

Given the weaker performance of aT2* < 20 ms at 1.5 T, a range of absolute T2* cut-offs were applied to determine the optimized T2* cut-offs that could identify comparable diagnostic performance and quantification of IMH as ssT2*. Figure 6 summarizes our findings and are detailed below for patients and animals separately.

*Patients* ROC analysis showed that the optimal cutoff at both acute and chronic phases MI is obtained when aT2* < 23 ms (p < 0.001 for both; see Fig. 6A). At this threshold, the sensitivity, specificity, accuracy and AUC for detecting IMH-positive segments were 90.2% (95% CI: 80.5–94.1), 96.7% (95% CI: 88.7–99.6), 92.8% (95% CI: 89.6–95.1), and 0.94 (95% CI: 0.90–0.96) in acute phase; and 90.5% (95% CI: 82.1–95.8), 92.5% (95% CI: 74.9–99.1), 91.0% (95% CI: 85.8–94.5), and 0.91 (95% CI: 0.85–0.96) in chronic phase. Based on whole-heart analysis, the total IMH volume quantified at the new cut-off was not different from that determined using ssT2* approach (Fig. 6B).

*Canines* ROC analysis showed that the optimal cutoff at both acute and chronic phases is obtained when aT2* < 25 ms (p < 0.001 for both; see Fig. 6C). At this threshold, the sensitivity, specificity, accuracy and AUC for detecting IMH-positive segments were 82.8% (95% CI: 64.2–94.2), 100% (95% CI: 73.5–100.0), 88.1% (95% CI: 77.2–94.4), and 0.91 (95% CI: 0.78–0.98) in acute phase, and 93.1% (95% CI: 77.2–99.2), 90.0% (95% CI: 68.3–98.8), 91.0% (95% CI: 81.5–96.2), and 0.92 (95% CI: 0.80–0.98) in chronic phase. Based on whole-heart analysis, the total IMH volume quantified at the new cut-off was not different from that determined using ssT2* approach (Fig. 6D).

### Relation to functional LV remodelling: subject-specific vs. absolute T2* thresholds

In patients, there was significant associations between ΔLVEDVI with acute IMH volume as measured with ssT2* and aT2* < 20 ms approaches (ssT2*: R^2^ = 0.34, p < 0.01; aT2* < 20 ms: R^2^ = 0.34, p < 0.01, Additional file 1: Fig. S3A), but there was no significant difference between the two approaches (p = 0.73). Similarly, significant correlations were found between ΔLVEF and acute IMH volume measured using both ssT2* and aT2* < 20 ms approaches (ssT2*: R^2^ = 0.37, p = 0.001; aT2* < 20 ms: R^2^ = 0.33, p < 0.01, See Additional file 1: Fig. S3C). While there was a difference in the ssT2* showing a stronger relation, this was not significantly different from than that observed with aT2* < 20 ms (p = 0.29). Neither ΔLVEDVI or ΔLVEF correlated with acute IMH T2* value. Refer to Additional file 1: Fig. S3 for additional details.

## Discussion

In one of the earliest publications in global myocardial iron overload, Anderson et al. demonstrated the utility of cardiac T2* mapping at 1.5T for noninvasively detecting abnormal myocardial iron using a T2* threshold of 20 ms [18]. Since then, cardiac T2* mapping has become the noninvasive standard for examining myocardial iron overload from hemochromatosis or transfusional siderosis [24], given that neither serum ferritin nor liver iron content gives a reliable assessment of myocardial iron overload, and cardiac biopsy is challenging [18, 20]. However, whether the 20-ms threshold adopted from the assessment of global iron overload conditions is optimal for detecting local accumulation of iron in the heart secondary to hemorrhagic MI is not known. Of further importance is the pathological underpinnings of hemorrhagic MIs are fundamentally different from those that lead to global myocardial iron overload. Despite these uncertainties, the absolute T2* threshold (aT2* < 20 ms) is still widely used to quantify the volume of IMH and concentration of iron (1/T2*) within the MI territories with IMH. In contrast, subject-specific T2* (ssT2*, using a mean-2SD criterion) has been validated against invasive standards [8, 9, 16] and used in several animal and clinical studies. However, there is a lack of understanding on whether the two approaches yield equivalent information.

To address this gap in knowledge, we investigated the concordance between ssT2* and aT2* < 20 ms approaches in patients and animal models of hemorrhagic MI at 1.5T. Both methods demonstrated excellent capacity for identification of hemorrhagic territories. However, we found that compared to ssT2* approach, aT2* < 20 ms lead to lower T2* value and volume of IMH in both patients and canines regardless of MI age. Our ex-vivo validation studies showed strong correlation between mass spectrometry and iron content in hemorrhagic tissue with both ssT2* and aT2* < 20 ms. Despite this, we found that the slope of the regression curves between T2* and iron concentration were significantly higher when using the ssT2* approach compared to the absolute threshold approach, independent of the actual threshold used with the methods. Thus our ex-vivo findings suggests that ssT2* would provide greater capacity for the identification of IMH as compared to absolute thresholds, especially when the hemorrhage is small.

Our findings here have implications for the diagnosis of hemorrhagic MI. In particular, given that ssT2* approach is likely to be more sensitive for identification of hemorrhagic MI, it offers the possibility to limit misdiagnosis of IMH in clinical settings. Most notably since IMH has emerged as an important prognostic predictor post MI, accurate identification of patients with IMH is expected to be important in the assessment of risk for adverse outcomes in the post MI setting. Further, our investigations into the optimized T2* cut-offs relative to ssT2* approach showed that a slightly higher T2* cut-off could potentially reduce the measured differences. These findings highlight the similarities and differences between the ssT2* and absolute T2* threshold approaches, and support the notion that the approaches cannot be interchangeably used. Our studies also showed that there is a significant relationship between relative changes in LVEDVI or LVEF between acute and chronic phases and IMH volume but not T2*. However, these relationships did not show dependence on the approach used to quantify IMH volumes. Thus, whether our findings suggest that smaller IMH volumes do not contribute to meaningful changes in LVEDVI or LVEF or is a reflection of inadequate statistical power of the current study requires further investigation.

Our study also shed some light on the evolution of hemorrhagic MI. Hemorrhagic byproducts, hemoglobin passes through several forms (namely oxyhemoglobin, deoxyhemoglobin, and methemoglobin) prior to red cell lysis and breakdown into ferritin and hemosiderin, which lead to T2* shortening in a progressively increasing manner [25]. Thus, a lower T2* caused by hemosiderin in the chronic phase as compared to acute phase would be expected. The animal data from acute to chronic phase in the present study conforms to this pathophysiological change at 8 weeks post MI. Unlike the animals, patients were studied at 6 month and exhibited a higher T2* value at chronic phase as compared to acute phase. This difference may stem from potential commencement of resolution of iron and/or other anatomical or biochemical changes in the infarct environment which may be confounded by imaging parameters associated with T2* acquisition, such as partial volume issues. Indeed Carberry et al. [26] reported approximately 40% reduction in IMH at 6 months post MI, but whether these differences are physiological reduction in iron or confounded by limitations in imaging requires further investigation.

### Limitations

Our study has limitations. First, our findings are limited to 1.5T. While 1.5T systems are most commonly used for CMR studies, additional studies are needed to extend our findings to 3T. Second, our validation is strictly limited to T2*, which as this study and many others in the field have demonstrated is highly correlated to the iron concentration. Validation of hemorrhage volume would have been useful but is not trivial as histological surface area measurements cannot be directly converted to volume measurements due to registration difficulties and the marked difference in spatial resolution in histology and CMR. Third, our validation was performed in the chronic setting. While there is a strong relationship between IMH in the acute phase being strongly correlated with chronic iron, additional studies invasive studies may be needed to validate our observations. Finally, our studies are limited to Siemens scanners operating at 1.5T. While these results are expected to hold across different vendor platforms, additional studies may be necessary to confirm whether our findings will hold up across all 1.5T scanner platforms. Finally it is anticipated that each field strength, T2* is likely impacted by choice imaging parameters, most notably spatial resolution. In the current study we employed standard scan parameters. With further advancement in T2*-weighted image acquisition, the influence of imaging parameters and their contribution for IMH detection and quantification need to be carefully considered.

## Conclusions

Currently used methods to quantify IMH, ssT2* and aT2* < 20 ms, have excellent capacity to identify IMH, albeit the T2*of IMH and volume of IMH based on aT2* < 20 ms are smaller compared to ssT2*. Thus the method used to quantify IMH from T2* CMR may influence the diagnosis of IMH.

## Supplementary Information

**Additional file 1: Figure S1.** Flow Diagram for Patient Studies. **Figure S2.** Flow Diagram for Pre-clinical Studies. **Table S1.** Comparison of correlation coefficients and slopes between T2* and IMH-Fe concentration between and within subject-specific, and absolute-threshold based approaches. **Figure S3.** Relationship between IMH Volume, T2* Value, and Functional LV Remodeling Using Subject-specific, and Absolute-threshold Based Approaches.

## Data Availability

The datasets used and/or analyzed during the current study are available from the corresponding author on reasonable request.

## Abbreviations

aT2*: Absolute T2*
AUC: Area under the curve
CI: Confidence interval
CMR: Cardiovascular magnetic resonance
ICC: Intraclass correlation coefficient
IMH: Intramyocardial hemorrhage
LGE: Late gadolinium enhancement
LV: Left ventricle/left ventricular
LVEDV: Left ventricular end-diastolic volume
LVEF: Left ventricular ejection fraction
LVESV: Left ventricular end-systolic volume
MI: Myocardial infarction
MVO: Microvascular obstruction
PCI: Percutaneous coronary intervention
PSIR: Phase sensitive inversion recovery
ROC: Receiver operating characteristics
ssT2*: Subject-specific T2*
STEMI: ST-segment elevation myocardial infarction
TTC: Triphenyl tetrazolium chloride
