# Correction to: Quantification of myocardial hemorrhage using T2* cardiovascular magnetic resonance at 1.5 T with ex-vivo validation

**DOI:** 10.1186/s12968-021-00821-5

**Published:** 2022-02-07

**Authors:** Yinyin Chen, Daoyuan Ren, Xingmin Guan, Hsin-Jung Yang, Ting Liu, Richard Tang, Hao Ho, Hang Jin, Mengsu Zeng, Rohan Dharmakumar

**Affiliations:** 1 Dept of Biomedical Sciences, Biomedical Imaging Research Institute, CedarsSinai Medical Center, Suite 400, 8700 Beverly Blvd, Los Angeles, CA 90048 USA; 2 Department of Radiology, Zhongshan Hospital, Fudan University, Shanghai, 200032 China; 3 Department of Cardiology, Zhongshan Hospital, Fudan University, Shanghai, China; 4 Department of Bioengineering, University of California at Los Angeles, Los Angeles, CA USA; 5 Department of Radiology, The First Afliated Hospital of China Medical University, Shenyang, 110001 China; 6 Academia Sinica, Taipei, Taiwan; 7 Department of Medical Imaging, Shanghai Medical School, Fudan University and Shanghai Institute of Medical Imaging, Shanghai, 200032 China

## Correction to: J Cardiovasc Magn Reson (2021) 23:104 https://doi.org/10.1186/s12968-021-00779-4

In the original publication [1] an error was introduced in the affiliations of Yinyin Chen due to a misunderstanding during the publication process. The incorrect and correct affiliations are listed below. The original article has been updated.

Correct affiliations:

- ^1^ Biomedical Imaging Research Institute, Cedars Sinai Medical Center, Los Angeles, CA
- ^2^ Department of Radiology, Zhongshan Hospital, Fudan University; Shanghai, 200032, China
- ^7^ Department of Medical Imaging, Shanghai Medical School, Fudan University and Shanghai Institute of Medical Imaging, Shanghai, 200032, China
  Incorrect affiliations:
- ^1^ Biomedical Imaging Research Institute, Cedars Sinai Medical Center, Los Angeles, CA
- ^2^ Department of Radiology, Zhongshan Hospital, Fudan University; Shanghai, 200032, China
- ^3^ Department of Cardiology, Zhongshan Hospital, Fudan University, Shanghai, China.
