# Supplementary material for: Quantification of myocardial hemorrhage using T2* cardiovascular magnetic resonance at 1.5T with ex-vivo validation
Source: J Cardiovasc Magn Reson. 2021 Sep 30;23:104. doi: 10.1186/s12968-021-00779-4 (PMC8482734; doi:10.1186/s12968-021-00779-4)
Supplement: Supplementary file 1 — Additional file 1: Figure S1. Flow Diagram for Patient Studies. Figure S2. Flow Diagram for Pre-clinical Studies. Table S1. Comparison of correlation coefficients and slopes between T2* and IMH-Fe concentration between and within subject-specific, and absolute-threshold based approaches. Figure S3. Relationship between IMH Volume, T2* Value, and Functional LV Remodeling Using Subject-specific, and Absolute-threshold Based Approaches. [file 12968_2021_779_MOESM1_ESM.docx]

**Figure S1** Flow Diagram for Patient Studies


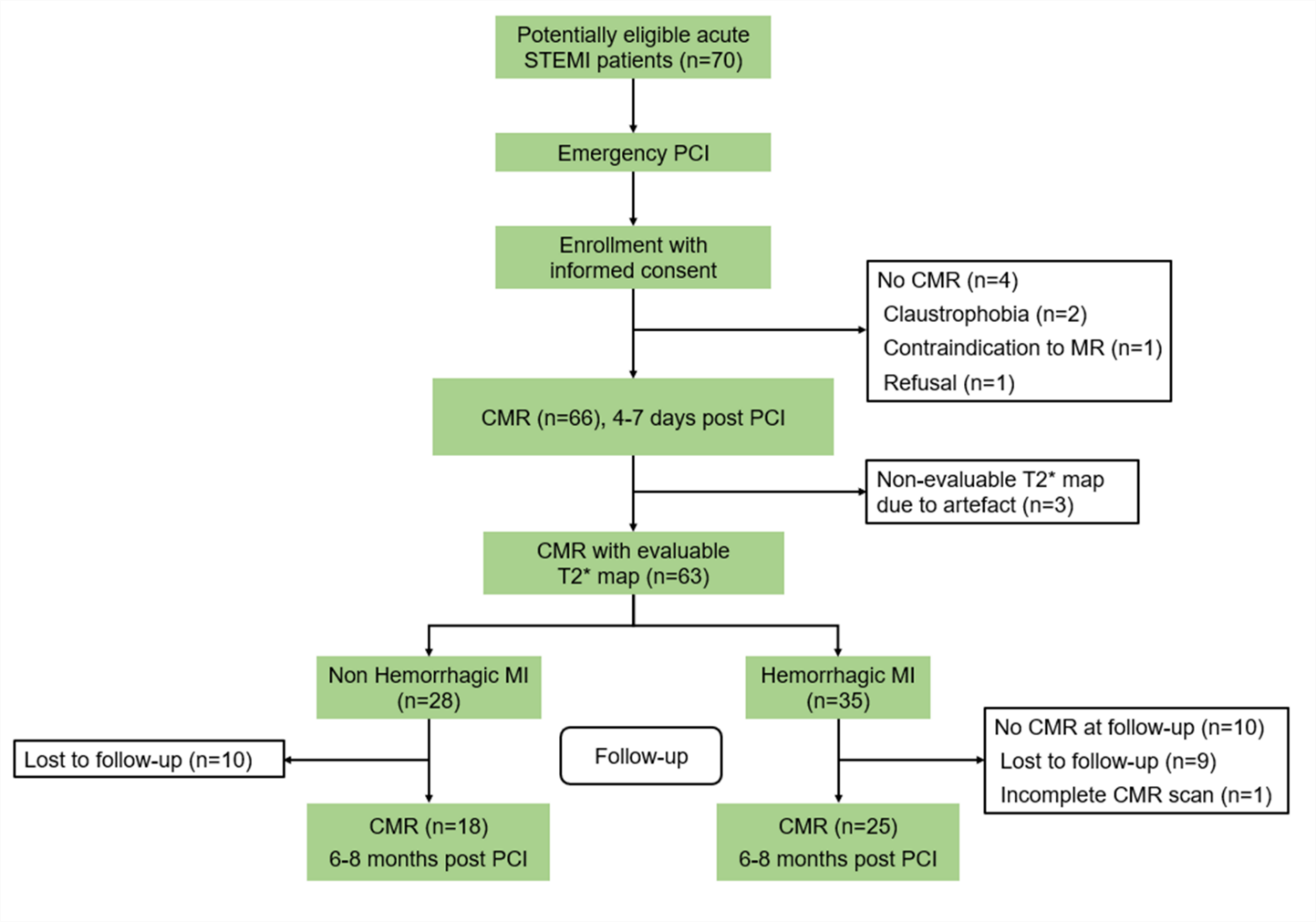


**Figure S2** Flow Diagram for Canines Studies


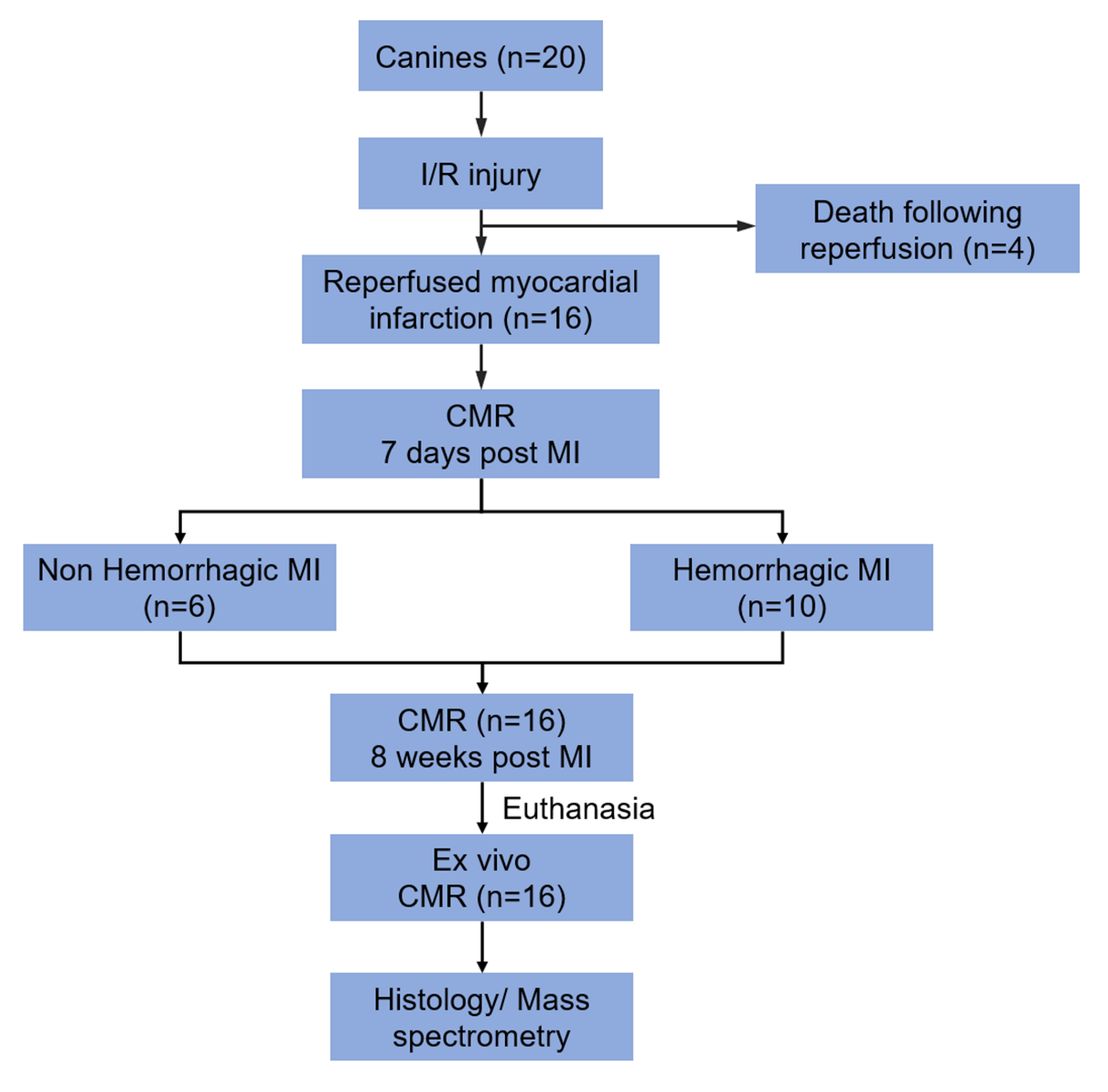


**Table S1** Comparison of Correlation Coefficients and Slopes between T2* and Iron Concentration Between and Within Subject-specific, and Absolute-threshold Based Approaches in Animals with IMH

| Log(T2*) /  -Log([Fe]_Hemo_) | Comparison of Pearson correlation coefficient (r) | p value | Comparison of slope | p value |
| --- | --- | --- | --- | --- |
| *Between subject-specific and absolute-threshold based approaches* | | | |  |
| 2SD vs 15ms | 0.97 vs 0.94 | 0.31 | 0.46 vs 0.29 | < 0.005* |
| 2SD vs 20ms | 0.97 vs 0.96 | 0.77 | 0.46 vs 0.32 | < 0.01* |
| 2SD vs 25ms | 0.97 vs 0.94 | 0.24 | 0.46 vs 0.32 | 0.02* |
| 3SD vs 15ms | 0.96 vs 0.94 | 0.54 | 0.45 vs 0.29 | < 0.01* |
| 3SD vs 20ms | 0.96 vs 0.96 | 1.00 | 0.45 vs 0.32 | 0.02* |
| 3SD vs 25ms | 0.96 vs 0.94 | 0.46 | 0.45 vs 0.32 | < 0.05* |
| 4SD vs 15ms | 0.95 vs 0.94 | 0.78 | 0.45 vs 0.29 | < 0.02* |
| 4SD vs 20ms | 0.95 vs 0.96 | 0.72 | 0.45 vs 0.32 | < 0.05* |
| 4SD vs 25ms | 0.95 vs 0.94 | 0.75 | 0.45 vs 0.32 | < 0.05* |
| *Subject-specific based approaches* | |  |  |  |
| 2SD vs 3SD | 0.97 vs 0.96 | 0.46 | 0.46 vs 0.45 | 0.87 |
| 2SD vs 4SD | 0.97 vs 0.95 | 0.30 | 0.46 vs 0.45 | 0.88 |
| 3SD vs 4SD | 0.96 vs 0.95 | 0.52 | 0.45 vs 0.45 | 0.99 |
| *Absolute-threshold based approaches* | |  |  |  |
| 15ms vs 20ms | 0.94 vs 0.96 | 0.37 | 0.29 vs 0.32 | 0.53 |
| 15ms vs 25ms | 0.94 vs 0.94 | 1.00 | 0.29 vs 0.32 | 0.53 |
| 20ms vs 25ms | 0.96 vs 0.94 | 0.45 | 0.32 vs 0.32 | 0.96 |

2SD, 3SD, and 4SD represent mean-2SD, mean-3SD, and mean-4SD respectively; 15ms, 20ms and 25ms represent aT2*<15ms, aT2*<20ms, and aT2*<25ms respectively

* indicates statistical significance (p< 0.05)


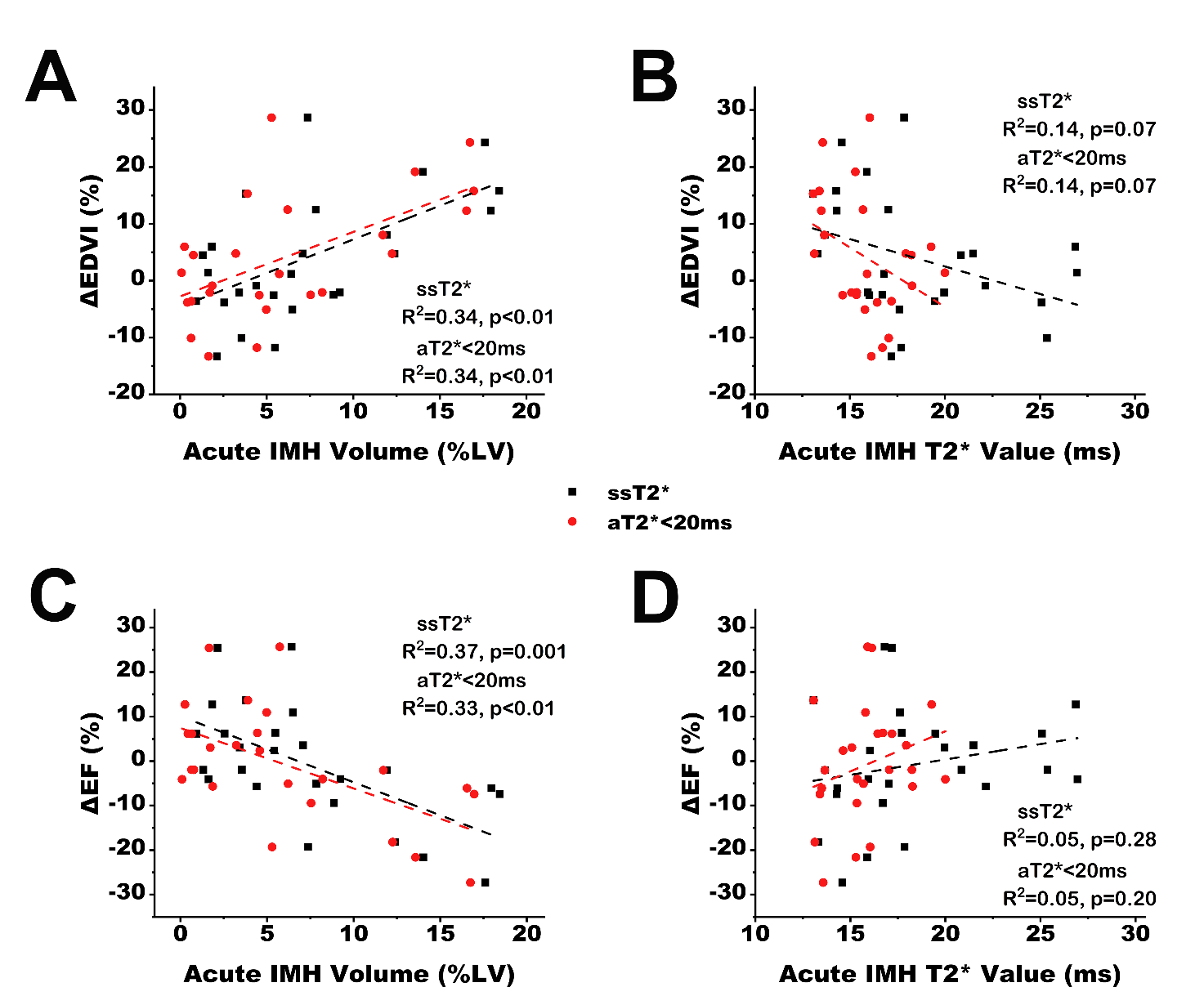


**Figure S3** Relationship between acute IMH Volume, T2* Value, and Functional LV Remodeling Based on ssT2* and aT2*<20ms Approaches in Patients with IMH. (A) ΔLVEDVI and acute IMH volume determined using both ssT2* and aT2*<20ms were significantly correlated. (B) was poorly associated with acute IMH T2*. (C) ΔLVEDVI and acute IMH volume determined using both ssT2* and aT2*<20ms were significantly correlated. (D) ΔLVEF was poorly associated with acute IMH T2*.
